# Idiopathic Intracranial Hypertension in a Child with Marfan Syndrome: Clinical, Neuroimaging, and Biomarker Findings from a Case Report

**DOI:** 10.3390/life16081292

**Published:** 2026-08-05

**Authors:** Giorgia Sforza, Carmen Maritato, Gaia Anzini, Alessia Carboni, Claudia Ruscitto, Laura Papetti, Massimiliano Valeriani

**Affiliations:** 1 Developmental Neurology Unit, Bambino Gesù Children’s Hospital IRCCS, 00165 Rome, Italy; claudia.ruscitto@opbg.net (C.R.); laura.papetti@opbg.net (L.P.); massimiliano.valeriani@opbg.net (M.V.); 2 Department of Maternal, Infantile and Urological Sciences, Sapienza University of Rome, 00185 Rome, Italy; 3 Fondazione Policlinico Universitario Campus Bio-Medico, 00128 Rome, Italy; 4 Diagnostic and Interventional Neuroradiology Unit and Vascular Anomalies, Bambino Gesù Children’s Hospital, IRCCS, 00165 Rome, Italy; alessia.carboni@opbg.net; 5 Systems Medicine Department, Hospital of Rome, Tor Vergata University, 00133 Rome, Italy; 6 Translational Pain Neuroscience and Precision Medicine, Center for Neuroplasticity and Pain, Department of Health Science and Technology, School of Medicine, Denmark University, 9220 Aalborg, Denmark

**Keywords:** idiopathic intracranial hypertension, Marfan syndrome, pediatrics, neurofilament light chain, papilledema, headache, IIH

## Abstract

Marfan syndrome (MFS) is a connective tissue disorder classically associated with cardiovascular, musculoskeletal, and ocular manifestations. Neurological involvement is increasingly recognized and is most commonly related to spontaneous intracranial hypotension secondary to dural ectasia and cerebrospinal fluid (CSF) leakage. By contrast, idiopathic intracranial hypertension (IIH) is exceptionally rare in pediatric patients with MFS. We report the case of an 8-year-old girl with Marfan syndrome presenting with neck pain, diplopia, bilateral papilledema, and bilateral sixth cranial nerve palsy. Brain MRI demonstrated optic nerve tortuosity, distension of the perioptic subarachnoid spaces, and partial empty sella, while venous sinus thrombosis and spinal CSF leakage were excluded. Lumbar puncture confirmed markedly elevated CSF opening pressure (48 cmH_2_O), consistent with IIH. CSF and plasma neurofilament light chain levels were elevated, whereas anti-MOG antibodies and autoimmune investigations were negative. The patient showed rapid clinical improvement following therapeutic CSF drainage and acetazolamide treatment. To the best of our knowledge, this represents only the second report of pediatric IIH associated with Marfan syndrome. Our patient developed idiopathic intracranial hypertension, an uncommon neurological manifestation in this condition. Through this case, we aim to highlight the diagnostic challenges, discuss the possible pathophysiological mechanisms underlying this rare association and emphasize the importance of considering intracranial hypertension in the differential diagnosis of children with Marfan syndrome presenting with neuro-ophthalmological symptoms.

## 1. Introduction

Marfan syndrome is a multisystem connective tissue disorder characterized by well-established cardiovascular, musculoskeletal, and ocular manifestations. In addition to these classical systemic features, involvement of the pulmonary, cutaneous, and central nervous systems has been increasingly recognized. Neurological abnormalities are frequently described, particularly in adult patients, and encompass a broad spectrum of structural and vascular complications [1,2,3]. Compared with the general population, individuals with Marfan syndrome are at increased risk of cerebrovascular abnormalities, including intracranial aneurysms, arterial dissections, and ischemic stroke [4].

Dural ectasia, one of the most frequent neuroradiological manifestations of Marfan syndrome, may predispose patients to cerebrospinal fluid (CSF) leakage, resulting in intracranial hypotension and typically presenting with orthostatic headache [5].

By contrast, the association between Marfan syndrome and idiopathic intracranial hypertension (IIH) remains poorly understood, particularly in the pediatric population. Here, we report a rare pediatric case of Marfan syndrome presenting with neuro-ophthalmological findings that ultimately led to the diagnosis of idiopathic intracranial hypertension.

**Case Report**

An 8-year-old girl with a clinically and genetically confirmed diagnosis of Marfan syndrome based on the revised Ghent criteria and identification of a pathogenic FBN1 variant was admitted for evaluation of diplopia, papilledema, and neck pain. At presentation, the patient’s auxological parameters were as follows: body weight 32 kg, height 142 cm and body mass index (BMI) 15.9 kg/m^2^, consistent with a normal nutritional status for age and sex. Her family history was notable for connective tissue disease: her mother had Marfan syndrome, while the maternal grandfather and maternal aunt had a history of mitral valve prolapse.

Approximately 20 days before admission, the patient experienced a brief febrile episode (maximum temperature 38.5 °C) lasting one day, followed by posterior neck pain without functional limitation, which resolved spontaneously within two days. Approximately one week later, she developed recurrent daily neck pain, worsening in the supine position and improving in the upright position, associated with asthenia, reduced appetite, and intermittent early-morning vomiting, in the absence of fever. These symptoms were initially interpreted as consistent with a viral illness, and no specific treatment was initiated.

One week later, the patient developed acute-onset left esotropia associated with diplopia, prompting admission to a regional hospital. Initial neuroimaging included brain computed tomography (CT) and brain magnetic resonance imaging (MRI) with magnetic resonance angiography (MRA). Brain CT revealed a right retrocerebellar arachnoid cyst. Brain MRI demonstrated mild asymmetry of the ocular globes, with slight elongation of the left anteroposterior axis suggestive of myopic buphthalmos, as well as tortuosity of the left optic nerve. No parenchymal signal abnormalities or pathological contrast enhancement were observed, and the intracranial arterial circulation appeared normal. Ophthalmological evaluation documented bilateral papilledema, more pronounced in the right eye. Orthoptic assessment revealed left esotropia with mild limitation of abduction. As no neurosurgical emergency was identified, the patient was discharged with oral ciprofloxacin for the treatment of a concomitant urinary tract infection. Before symptom onset, she had not been exposed to medications classically associated with secondary intracranial hypertension, including tetracyclines, retinoids (vitamin A derivatives), corticosteroids, recombinant human growth hormone, or corticosteroid withdrawal. Ciprofloxacin therapy was initiated only after the onset of neurological symptoms and therefore was considered unlikely to have contributed to the development of intracranial hypertension.

Ten days later, because of persistent diplopia, new-onset deviation of the right eye in addition to the pre-existing left ocular deviation, and ongoing neck pain, the patient presented again to the emergency department. Orthoptic examination demonstrated a 40–prism diopter esotropia at both near and distance fixation, associated with homonymous diplopia, marked bilateral lateral rectus muscle hypofunction, impaired elevation (more pronounced on the left side), and bilateral papilledema. Visual acuity was preserved. Color Doppler ultrasonography excluded carotid artery dissection. Neurological examination revealed bilateral sixth cranial nerve palsy, gaze-evoked nystagmus at extreme gaze, and persistent neck pain in the absence of motor deficits; gait was cautious but autonomous. In light of the overall clinical presentation, the patient was admitted to the Developmental Neurology Unit for further diagnostic evaluation.

**Figure 1:**
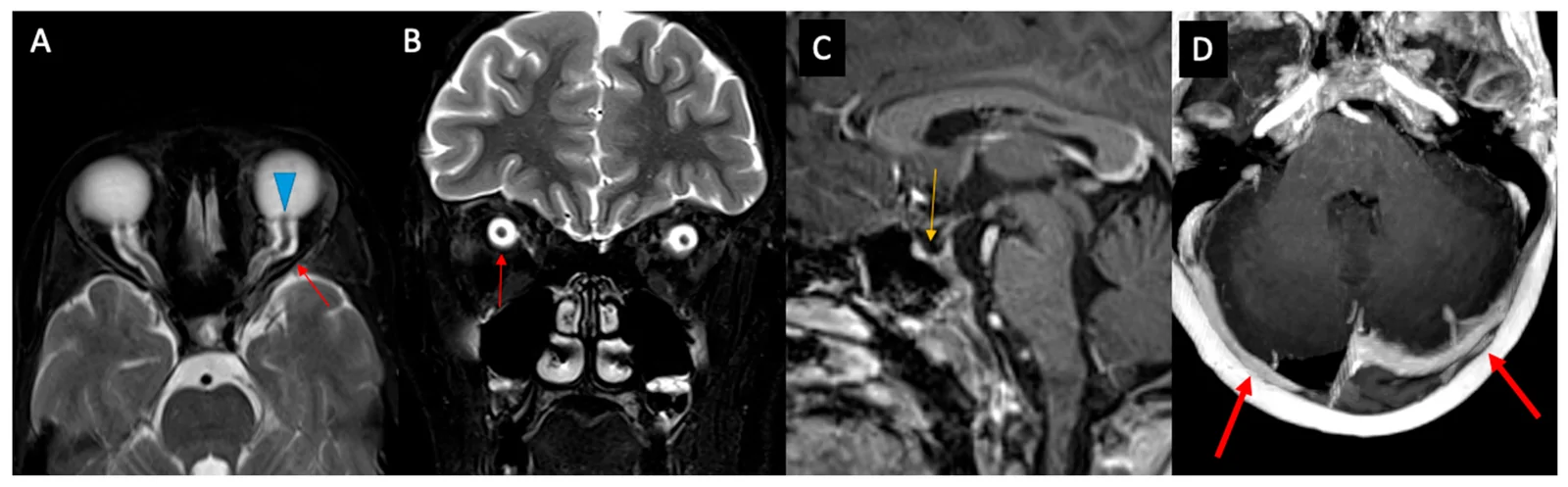
Brain and orbital MRI performed on a 3-T scanner, including standard brain sequences with 3 mm slice thickness and dedicated high-resolution orbital sequences with 1 mm slice thickness. T2- and T1-weighted magnetic resonance imaging (MRI) sequences demonstrating indirect radiological signs of intracranial hypertension. Axial T2-weighted (**A**) and coronal fat-saturated T2-weighted (**B**) images show bilateral distension of the perioptic subarachnoid spaces (red arrows) and bilateral optic disc protrusion (blue arrowheads). Contrast-enhanced T1-weighted image (**C**) demonstrates herniation of the suprasellar cistern into the sella turcica with compression of the pituitary gland, consistent with partial empty sella (yellow arrowhead). The axial contrast-enhanced T1-weighted image (**D**) also demonstrates patent transverse venous sinuses with mild indentation, without evidence of venous thrombosis.

In light of the clinical suspicion of idiopathic intracranial hypertension and to exclude cerebrovascular abnormalities known to be associated with Marfan syndrome, brain MRI, magnetic resonance angiography (MRA), and whole-spine MRI were performed. These investigations confirmed the previously described findings and additionally demonstrated herniation of the suprasellar cistern into the sella turcica, consistent with an empty sella sign. The optic nerves appeared markedly tortuous, with distension of the perioptic subarachnoid spaces and bilateral optic disc prominence (Figure 1). The presence of venous sinus thrombosis was excluded. No spinal cord abnormalities or dural ectasia were identified.

The retrocerebellar arachnoid cyst was considered an incidental finding, as it showed no mass effect or evidence of cerebrospinal fluid pathway obstruction and was therefore deemed unrelated to the patient’s intracranial hypertension.

Based on the presence of papilledema, bilateral sixth cranial nerve palsy, characteristic neuroradiological findings, and the absence of secondary causes on neuroimaging, idiopathic intracranial hypertension (IIH) was suspected. Lumbar puncture, performed in the lateral decubitus position, revealed clear cerebrospinal fluid (CSF) with an elevated opening pressure of 48 cmH_2_O. Therapeutic CSF drainage was performed until a closing pressure of 23 cmH_2_O was achieved, supporting the diagnosis of intracranial hypertension.

**Figure 2:**
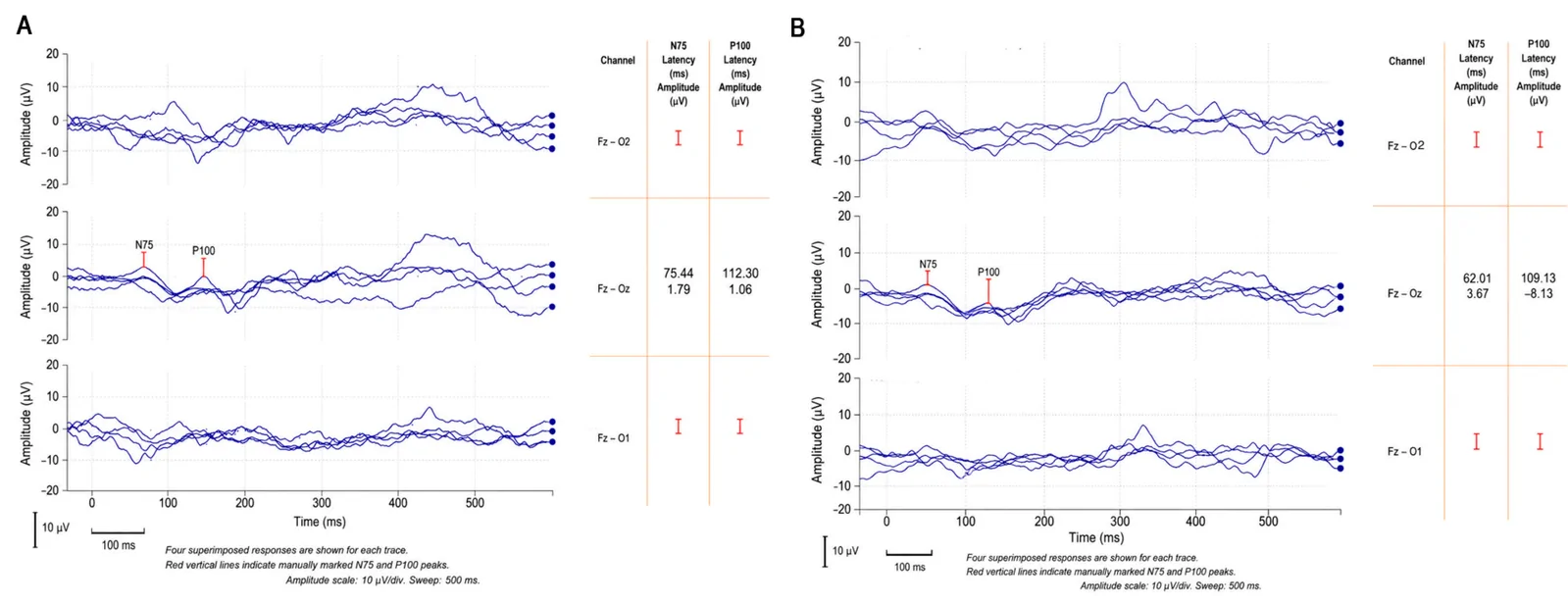
Pattern-reversal visual evoked potentials (30′) recorded from the left eye (**A**) and right eye (**B**), demonstrating symmetrical cortical P100 responses with a physiological double-peak morphology and latency values within the normal range for age. The P100 marker was positioned at the midpoint of the double-peak complex to account for this physiological variant. Flash electroretinography (flash-ERG) was also normal bilaterally. To complete the evaluation, a study of retinal evoked potentials (flash-ERG) was also performed; the results were normal and symmetrical (they are not reported due to their limited specific diagnostic value in cases of intracranial hypertension). VEPs were recorded using a Micromed System Plus® system (Micromed S.p.A., Mogliano Veneto, Italy) according to IFCN recommendations. Recordings were obtained with a 500 ms analysis window, a sensitivity of 10 μV/div, and standard band-pass filter settings.

The study of pattern-reversal visual evoked potentials (VEPs) using a 30′ check size revealed symmetrical cortical P100 responses with a peculiar double-peak morphology (physiological variant), and P100 latencies were within the normal range for age (Figure 2). In accordance with this waveform morphology, the P100 latency marker was positioned at the midpoint of the double-peaked positive complex to ensure consistent identification of the principal response. Recordings were obtained using a band-pass filter of 0.1–100 Hz, a 500 ms analysis time, and a sensitivity of 10 μV/div.

Cerebrospinal fluid analysis, including chemical–physical and cytological examination, was unremarkable. Neurofilament light chain (NfL) concentrations were markedly elevated in both cerebrospinal fluid (5807 pg/mL; reference value <500 pg/mL) and plasma (94 pg/mL; reference value <20 pg/mL), corresponding to 11.6-fold and 4.7-fold increases above the upper reference limit, respectively. NfL concentrations were measured using the Human Simple Plex assay (ProteinSimple, San Jose, CA, USA) on the Ella™ automated immunoassay platform (ProteinSimple, San Jose, CA, USA), according to the manufacturer’s instructions.

Additional blood investigations, including anti-myelin oligodendrocyte glycoprotein (MOG) antibodies, vitamin profile assessment, and hormonal evaluation (thyroid function and parathyroid hormone levels), were all within normal limits. Autoimmune screening and CSF studies excluded secondary etiologies. The main cerebrospinal fluid findings and relevant laboratory investigations are summarized in Table 1.

Treatment with acetazolamide (15 mg/kg/day) was initiated, together with oral bicarbonate supplementation. Following lumbar puncture and therapeutic CSF drainage, the patient showed immediate clinical improvement, with marked reduction of headache and neck pain, as well as partial improvement of the bilateral sixth cranial nerve palsy. Progressive further improvement was observed during hospitalization.

Given the underlying diagnosis of Marfan syndrome, a cardiological evaluation was also performed. Electrocardiography demonstrated sinus bradycardia, while echocardiography revealed mild mitral valve prolapse with trivial regurgitation.

After seven days of hospitalization, the patient was discharged in good clinical condition with a diagnosis of idiopathic intracranial hypertension associated with Marfan syndrome. Outpatient follow-up was arranged at a specialized pediatric headache center and the pediatric cardiology unit. At the first post-discharge neurological follow-up visit, she reported complete resolution of headache, neck pain, and diplopia, while neurological examination confirmed complete recovery from the bilateral sixth cranial nerve palsy.

Serial follow-up evaluations documented sustained clinical improvement. Fundus examinations performed in January and April 2026 demonstrated complete resolution of papilledema. Optic nerve ultrasonography performed in May 2026 revealed normal optic nerve sheath diameters bilaterally, with no evidence of perioptic cerebrospinal fluid accumulation. The patient remained asymptomatic throughout follow-up while receiving acetazolamide and bicarbonate therapy. During treatment, serial blood gas analyses and serum electrolyte measurements were performed to monitor for acetazolamide-related metabolic acidosis and electrolyte disturbances; no clinically significant abnormalities were detected. In light of the complete clinical, ophthalmological, and ultrasonographic resolution, acetazolamide and bicarbonate therapy was gradually tapered and discontinued after approximately seven months of treatment.

T2- and T1-weighted magnetic resonance imaging (MRI) sequences demonstrating indirect radiological signs of intracranial hypertension. Axial T2-weighted (A) and coronal fat-saturated T2-weighted (B) images show bilateral distension of the perioptic subarachnoid spaces (red arrows) and bilateral optic disc protrusion (blue arrowheads). Contrast-enhanced T1-weighted image (C) demonstrates herniation of the suprasellar cistern into the sella turcica with compression of the pituitary gland, consistent with partial empty sella. The axial contrast-enhanced T1-weighted image (D) also demonstrates patent transverse venous sinuses with mild indentation, without evidence of venous thrombosis.

## 2. Discussion

Marfan syndrome (MFS) is an autosomal dominant connective tissue disorder caused by pathogenic variants in *FBN1*, characterized by cardiovascular, musculoskeletal, and ocular involvement [1,2]. Neurological manifestations are increasingly recognized and are mainly related to vascular tortuosity and connective tissue fragility affecting the meninges and dural structures [3,4]. The most frequently reported neurological symptom is migraine-like headache, typically without aura, which has been associated with cerebrovascular tortuosity and altered vascular compliance [3,4].

Orthostatic headache is another well-described neurological manifestation and is most commonly related to spontaneous intracranial hypotension secondary to dural ectasia and cerebrospinal fluid (CSF) leakage [5,6,7,8]. Dural ectasia, in particular, represents one of the hallmark neuroradiological features of MFS and may predispose patients to chronic CSF loss through meningeal fragility [8]. Consequently, intracranial hypotension has emerged as the predominant CSF pressure phenotype described in both adult and pediatric patients with MFS [9,10,11].

By contrast, intracranial hypertension in patients with Marfan syndrome appears to be exceedingly rare, particularly in childhood, with only isolated pediatric cases reported in the literature [12]. The association is somewhat paradoxical, as patients with Marfan syndrome are typically characterized by a lean body habitus and do not exhibit the obesity that represents one of the strongest risk factors for idiopathic intracranial hypertension. As a result, raised intracranial pressure may not be immediately suspected in these patients, potentially leading to delayed diagnosis. This observation further supports the hypothesis that connective tissue abnormalities may contribute to CSF pressure dysregulation through mechanisms distinct from those involved in classical obesity-related IIH.

Recent literature has proposed that connective tissue abnormalities involving collagen, elastin, extracellular matrix architecture, venous compliance, and meningeal integrity may lead to dysregulation of CSF dynamics, resulting in a spectrum of pressure phenotypes ranging from intracranial hypotension to intracranial hypertension [13]. Although these mechanisms have been primarily investigated in Ehlers–Danlos syndromes and related hypermobility disorders, they may also provide a plausible pathophysiological framework for atypical CSF pressure regulation in Marfan syndrome. Altered biomechanical properties of the dura and venous system could theoretically impair CSF absorption and venous drainage, thereby contributing to intracranial hypertension in susceptible individuals.

Our patient presented with a clinical and neuroradiological profile highly suggestive of idiopathic intracranial hypertension, including papilledema, bilateral sixth cranial nerve palsy, optic nerve tortuosity, distension of the perioptic subarachnoid spaces, and partial empty sella. Importantly, repeat neuroimaging excluded secondary causes of raised intracranial pressure, including venous sinus thrombosis, venous sinus stenosis, hydrocephalus, intracranial mass lesions, and vascular complications potentially associated with Marfan syndrome. Moreover, whole-spine MRI did not reveal dural ectasia or evidence of spinal CSF leakage, making a low-pressure CSF state unlikely and supporting a true hypertensive CSF profile, subsequently confirmed by lumbar puncture with markedly elevated opening pressure.

An additional notable finding in our case was the marked elevation of neurofilament light chain (NfL) levels in both CSF and plasma. NfL is increasingly recognized as a biomarker of neuroaxonal injury and has recently been investigated in idiopathic intracranial hypertension [14]. A recent systematic review reported altered CSF and serum biomarker profiles in IIH, including elevated NfL levels, potentially reflecting axonal stress and optic nerve injury secondary to chronically elevated intracranial pressure [14]. In our patient, the marked increase in CSF NfL levels may therefore represent evidence of subclinical neuroaxonal involvement related to raised intracranial pressure and papilledema.

Furthermore, anti-myelin oligodendrocyte glycoprotein (MOG) antibodies were assessed because isolated intracranial hypertension has recently been described as a possible presenting manifestation of MOG antibody-associated disease (MOGAD), particularly in pediatric patients [15].

The patient showed rapid clinical improvement following therapeutic CSF drainage, with immediate reduction of headache and neck pain and partial recovery of bilateral sixth cranial nerve palsy, followed by progressive improvement during acetazolamide therapy. This favorable response further supports the diagnosis of IIH and underlines the importance of early recognition and treatment to prevent permanent visual sequelae.

An interesting clinical observation in our patient is the history of a self-limited febrile illness occurring approximately 20 days before the onset of neurological symptoms. Although a causal relationship cannot be established, previous reports have suggested that viral infections may act as potential triggers of intracranial hypertension in children through transient inflammatory or immune-mediated mechanisms affecting cerebrospinal fluid dynamics. A pediatric case series has suggested that viral infections may act as precipitating factors for intracranial hypertension in susceptible individuals, and isolated cases following varicella infection have also been reported [16,17]. In our patient, the temporal association between the febrile episode and the subsequent development of idiopathic intracranial hypertension raises the possibility that the preceding infection may have acted as a triggering event in the setting of an underlying connective tissue disorder. However, given the lack of microbiological confirmation and the observational nature of this case, this hypothesis remains speculative.

This report has several limitations. As a single case, it does not allow generalization of the findings or establish a causal relationship between Marfan syndrome and idiopathic intracranial hypertension. In addition, despite complete clinical, ophthalmological, and radiological recovery, longer-term follow-up is required to assess the risk of recurrence. Finally, the clinical significance of elevated neurofilament light chain concentrations in pediatric idiopathic intracranial hypertension remains to be established in larger studies.

In conclusion, this case expands the limited evidence linking Marfan syndrome with pediatric idiopathic intracranial hypertension and highlights the importance of considering raised intracranial pressure in children with connective tissue disorders presenting with neuro-ophthalmological symptoms, even in the absence of obesity or dural ectasia. The marked elevation of neurofilament light chain also suggests a potential role for this biomarker in identifying neuroaxonal injury associated with pediatric idiopathic intracranial hypertension, warranting further investigation.

## 3. Conclusions

This case highlights the importance of considering intracranial hypertension in the differential diagnosis of pediatric patients with Marfan syndrome presenting with visual disturbances, headache, or cranial nerve palsies. Reliance on the traditional association between Marfan syndrome and low-CSF-pressure states may result in diagnostic delay and under-recognition of hypertensive CSF disorders.

To the best of our knowledge, this represents only the second report in the literature describing idiopathic intracranial hypertension in a pediatric patient with Marfan syndrome, further emphasizing the rarity of this association.

Careful neuro-ophthalmological evaluation, dedicated neuroradiological assessment, and direct measurement of CSF opening pressure are essential for accurate diagnosis. In selected pediatric cases, ancillary investigations such as anti-MOG antibody testing and neurofilament light chain measurement may provide additional diagnostic and pathophysiological insights, particularly when inflammatory or neuroaxonal mechanisms are suspected. Early diagnosis and prompt treatment remain crucial to prevent irreversible visual complications.

## Figures and Tables

**Table 1:** Summary of cerebrospinal fluid and relevant laboratory findings.

Parameter	Result	Reference Range
***Cerebrospinal fluid (CSF) analysis***		
Appearance	Clear	Clear
Color	Colorless	Colorless
Opening pressure *	48 cmH_2_O	<28 cmH_2_O
White blood cells	2 cells/mm^3^	0–5 cells/mm^3^
Red blood cells	10 cells/mm^3^	0 cells/mm^3^
Total protein	17 mg/dL	10–43 mg/dL
Glucose	58 mg/dL	60–80 mg/dL
Albumin	6 mg/dL	14–35 mg/dL
Lactate	1.83 mmol/L	1.10–2.80 mmol/L
CSF cytology	Negative for atypical cells	Negative
***Additional investigations***		
Intact parathyroid hormone (PTH)	35.3 pg/mL	15.0–65.0 pg/mL
Anti-MOG antibodies	Negative	Negative
CSF neurofilament light chain (NfL)	5807 pg/mL	<500 pg/mL
Plasma neurofilament light chain (NfL)	94 pg/mL	<20 pg/mL

* Lumbar puncture performed in the lateral decubitus position.

## Data Availability

Since this is a single-patient case report, the data are not publicly available due to privacy and ethical restrictions. De-identified data may be made available from the corresponding author upon reasonable request, subject to institutional and ethical approval.
